# Artificial Intelligence–Based Methods for Integrating Local and Global Features for Brain Cancer Imaging: Scoping Review

**DOI:** 10.2196/47445

**Published:** 2023-11-17

**Authors:** Hazrat Ali, Rizwan Qureshi, Zubair Shah

**Affiliations:** 1 College of Science and Engineering Hamad Bin Khalifa University Doha Qatar; 2 Department of Imaging Physics MD Anderson Cancer Center University of Texas, Houston Houston, TX United States

**Keywords:** artificial intelligence, AI, brain cancer, brain tumor, medical imaging, segmentation, vision transformers

## Abstract

**Background:**

Transformer-based models are gaining popularity in medical imaging and cancer imaging applications. Many recent studies have demonstrated the use of transformer-based models for brain cancer imaging applications such as diagnosis and tumor segmentation.

**Objective:**

This study aims to review how different vision transformers (ViTs) contributed to advancing brain cancer diagnosis and tumor segmentation using brain image data. This study examines the different architectures developed for enhancing the task of brain tumor segmentation. Furthermore, it explores how the ViT-based models augmented the performance of convolutional neural networks for brain cancer imaging.

**Methods:**

This review performed the study search and study selection following the PRISMA-ScR (Preferred Reporting Items for Systematic Reviews and Meta-Analyses extension for Scoping Reviews) guidelines. The search comprised 4 popular scientific databases: PubMed, Scopus, IEEE Xplore, and Google Scholar. The search terms were formulated to cover the interventions (ie, ViTs) and the target application (ie, brain cancer imaging). The title and abstract for study selection were performed by 2 reviewers independently and validated by a third reviewer. Data extraction was performed by 2 reviewers and validated by a third reviewer. Finally, the data were synthesized using a narrative approach.

**Results:**

Of the 736 retrieved studies, 22 (3%) were included in this review. These studies were published in 2021 and 2022. The most commonly addressed task in these studies was tumor segmentation using ViTs. No study reported early detection of brain cancer. Among the different ViT architectures, Shifted Window transformer–based architectures have recently become the most popular choice of the research community. Among the included architectures, UNet transformer and TransUNet had the highest number of parameters and thus needed a cluster of as many as 8 graphics processing units for model training. The brain tumor segmentation challenge data set was the most popular data set used in the included studies. ViT was used in different combinations with convolutional neural networks to capture both the global and local context of the input brain imaging data.

**Conclusions:**

It can be argued that the computational complexity of transformer architectures is a bottleneck in advancing the field and enabling clinical transformations. This review provides the current state of knowledge on the topic, and the findings of this review will be helpful for researchers in the field of medical artificial intelligence and its applications in brain cancer.

## Introduction

### Background

Brain cancer is typically characterized by a brain tumor. A brain tumor is a mass or development of aberrant brain cells. The signs and symptoms of a brain tumor vary widely and are determined by the size, location, and rate of growth of the brain tumor. Brain tumors can originate in the brain (primary brain tumors) or move from other body regions to the brain (secondary or metastatic brain tumors). In general, studying brain cancer is challenging given the highly complex anatomy of the human brain, where several sections are responsible for various nervous system processes [1].

Medical imaging technologies for studying the brain are rapidly advancing. Therefore, it is critical to provide tools to extract information from brain image data such that they may aid in automatic or semiautomatic computer-aided diagnosis of brain cancer. Artificial intelligence (AI) techniques based on modern machine learning and deep learning models enable computers to make data-driven predictions using massive amounts of data. These techniques have a wide range of applications, many of which can be customized to extract useful information from medical images [2-6].

Among AI techniques developed for brain cancer applications, architectures based on convolutional neural networks (CNNs) have dominated the research on brain cancer diagnosis and classification. For example, UNet (an encoder-decoder CNN architecture) and its variants [7,8] are popular for brain tumor segmentation tasks. However, CNNs are known to be effective in extracting only local dependencies in the input image data, which is mainly attributed to the localized receptive field. Compared with CNNs, attention-based transformer models (transformers) [9] are good at capturing long-range dependencies. Given their ability to learn long-range dependencies, transformers form the backbone of most state-of-the-art models in the natural language processing domain [10].

For image classification tasks, Dosovitskiy et al [11] proposed the computer vision variants of the transformer architecture, typically known as vision transformer (ViT). The concept of attention was applied to images by representing them as a sequential combination of 16×16-pixel patches. The image patches were processed in a way similar to tokens (words) in natural language processing [11]. The sections (with positional embeddings) are ordered. The embeddings are vectors that can be learned. Each piece is organized in a straight line and multiplied by the embedding matrix. The position embedding result is passed to the transformer encoder.

Given the potential demonstrated by transformer-based approaches for computer vision tasks, transformers have quickly penetrated the field of medical imaging. For example, some studies [12-15] have used them on computed tomography scans and x-ray images of the lungs to classify COVID-19 and pneumonia. Similarly, Zhang and Zhang [16] and Xie et al [17] used ViT for medical image segmentation, and He et al [18] used ViT for brain age estimation. With the recent developments of ViTs in computer vision applications, there has been a growing interest in developing ViT-based architectures for cancer imaging applications. ViT can also aid in the diagnosis and prognosis of other types of cancers. For example, Chen et al [19] showed the scaling of ViTs to large whole-slide imaging for 33 different cancer types. The benchmarking results demonstrate that the transformer-based architecture with hierarchical pretraining outperforms the existing cancer subtyping and survival prediction methods, indicating its effectiveness in capturing the hierarchical phenotypic structure in tumor microenvironments.

Accordingly, many recent efforts have been reported on the developments of ViT architectures to make progress in brain cancer applications. With the growing interest in developing ViT-based methods for brain cancer imaging, there is a dire need to review the recent developments and identify the key challenges. To the best of our knowledge, no study (review) has reported the different ViT architectures for brain cancer imaging and analyzed how ViT complements CNNs in brain cancer diagnosis, classification, grading, and brain tumor segmentation.

A few review and survey articles that are relevant to our work are by Parvaiz et al [20], Magadza and Viriri [21], Akinyelu et al [22], He et al [23], and Biratu et al [24]. Among these, Magadza and Viriri [21] and Biratu et al [24] have surveyed the articles that used deep learning and machine learning methods for brain tumor segmentation. In addition, they covered papers until mid-2021 only and did not cover studies on ViT. Similarly, the survey by Akinyelu et al [22] has a broad scope, as it covered different methods including CNNs, capsule networks, and ViT used for brain tumor segmentation. In addition, it included only 5 studies on ViT, of which 4 were from 2022. Reviews by Parvaiz et al [20], He et al [23], and Shamshad et al [25] covered the applications of ViT in medical imaging; however, the scope of all these reviews is broad, as they included different medical imaging applications. In addition, they conducted a descriptive study of ViT for various medical imaging modalities. Similarly, many relevant recent studies on ViT-based architectures have been left out, as both the reviews [20,25] were released in early 2022. Nevertheless, the aforementioned reviews could be of interest to the readers. Table 1 compares our review with the previously published review articles.

Compared with other existing reviews on ViTs and medical imaging, our study is specific to brain cancer applications and covers the most recent developments. This review provides quantitative insights into the computational complexity and the required computational resources to implement ViT architectures for brain cancer imaging. Such insights will be helpful for the researchers to choose hardware resources and graphics processing units (GPUs). This review identifies the research challenges that are specific to ViT-based approaches in brain cancer imaging applications. These discussions will raise awareness for the related research directions. This review identifies the available public data sets and highlights the need for additional data to motivate the community to develop more publicly available data sets for brain cancer research. Furthermore, this review follows a narrative synthesis approach that would help the readers follow the text quickly.

**Table 1:** Comparison with similar review articles.

Review title	Month and year	Scope and coverage	Comparison with our review
Vision transformers in Medical Computer Vision—A Contemplative Retrospection [20]	March 2022	The title is specific to ViT^a^; however, the full text has a very broad scope with discussions on deep learning, CNNs^b^, and ViT. It covers different applications in medical computer vision, including the classification of disease, segmentation of tissues, registration tasks in medical images, and image-to-text applications. It does not provide much text on brain cancer applications of ViT. Many recent studies of 2022 are left out as the preprint was released in March 2022. It does not provide a comparative study on the computational complexity of ViT-based models.	Our review is also specific to ViT. Our review is specific to brain cancer applications. Our review includes more recent studies on ViT. Our review provides a comparative study of the computational complexity of the ViT-based models.
Transformers in medical imaging: A survey [25]	January 2022	It is specific to ViT. It has a broad scope as different medical imaging applications are included. It does not include many recent studies on ViT for brain cancer imaging (as the preprint was released in January 2022).	Our review is also specific to ViT. Our review is specific to brain cancer applications. Our review includes more recent studies on ViT.
Transformers in Medical Image Analysis: A Review [23]	August 2022	It is specific to ViT. It has broad scope as different medical imaging applications are included. It provides a descriptive review of ViT techniques for different medical imaging modalities. It does not provide a quantitative analysis of the computational complexity of ViT-based methods.	Our review is also specific to ViT. Our review is specific to brain cancer applications. Our review provides a comparative study of the computational complexity of the ViT-based models.
Brain Tumor Diagnosis Using Machine Learning, Convolutional Neural Networks, Capsule Neural Networks and Vision Transformers, Applied to MRI^c^: A Survey [22]	July 2022	It covers applications specific to brain tumor segmentation. It has a broad scope, as it includes studies on CNNs, capsule networks, and ViT. It includes only 5 studies on ViT. Many recent studies are left out as it covers only 4 studies from 2022. It provides no quantitative analysis of computational complexity.	Our review is also specific to brain cancer and brain tumor. Our review covers more recent studies. Our review includes 22 studies on ViT for brain cancer application. Our review provides a comparative study of the computational complexity of the ViT-based models.
A survey of brain tumor segmentation and classification algorithms [24]	September 2021	It has a very broad scope as it covers traditional machine learning and deep learning methods. It covers studies until early 2021 only.	Our review is specific to ViT. Our review covers more recent studies.
Deep learning for brain tumor segmentation: a survey of state-of-the-art [21]	January 2021	It has a broad scope as it covers different deep learning methods. Many recent studies are left out.	Our review is specific to ViT. Our review covers more recent studies.

^a^ViT: vision transformer.

^b^CNN: convolutional neural network.

^c^MRI: magnetic resonance imaging.

### Research Problem

The popularity of transformer-based approaches for medical imaging has been increasing. Many recent studies have developed new transformer-based methods for brain cancer application. Hence, there is a need to review the recent studies on how transformer-based approaches have contributed to brain cancer diagnosis, grading, and tumor segmentation. In this study, we present a review of the advancements in ViTs for brain cancer imaging applications. We present the recent ViT architectures for brain cancer diagnosis and classification, identify the key pipelines for combining ViT with CNNs, and highlight the key challenges and issues in developing ViT-based AI techniques for brain cancer imaging. More specifically, this review aims to identify the common techniques that were developed to use ViT for brain tumor segmentation and whether ViTs were effective in enhancing the segmentation performance. This review also identifies the common modality of brain imaging data used for training ViT for brain tumor segmentation. Moreover, this review identifies the commonly used data sets for the brain tumor that contributed to developing ViT-based models. Finally, this review presents the key challenges that the researchers faced in developing ViT-based approaches for brain tumor segmentation. We believe that this review will help researchers in deep learning and medical imaging interdisciplinary fields to understand the recent developments on the topic. Furthermore, it will appeal students and researchers interested to know about the advancements in brain cancer imaging.

## Methods

### Overview

We performed a literature search in famous scientific databases and conducted a scoping review following the PRISMA-ScR (Preferred Reporting Items for Systematic Reviews and Meta-Analyses extension for Scoping Reviews) guidelines [26]. Multimedia Appendix 1 provides the PRISMA-ScR checklist. The literature search and the study selection were performed using the steps described in the following subsections.

### Search Strategy

#### Search Sources

We searched for relevant literature in 4 databases: PubMed, Scopus, IEEE Xplore, and Google Scholar. The search was performed between July 31 and August 1, 2022. For Google Scholar, we retained the first 300 results, as the results beyond 300 lacked relevance to the topic of this review. We also screened the reference lists of the included studies to retrieve any additional studies that fulfilled the inclusion criteria.

#### Search Terms

We defined the key terms for the search by referring to the available literature and by a discussion with domain experts. The search terms comprised the terms corresponding to the intervention (ie, transformers) and the target application (ie, cancer and tumor). The search strings are provided in Multimedia Appendix 2.

### Search Eligibility Criteria

Our search focused on studies that reported developing ViT-based architectures for brain tumor segmentation, brain cancer diagnosis, or prognosis. We considered studies conducted between January 2017 and July 2022. We included studies that used ViT with or without combining other deep learning architectures, such as CNN, and excluded studies that used only CNN. We excluded studies that reported the diagnosis of other cancer types, such as lung cancer or colorectal cancer, and did not report the use of the model for any form of brain cancer. We included studies that used any type of brain cancer data, including brain magnetic resonance imaging (MRI) and histopathology image data. We included studies published as peer-reviewed articles or conference proceedings and excluded nonpeer-reviewed articles (preprints), short notes, editorial reviews, abstracts, and letters to the editor. We excluded survey and review articles. We did not impose any additional restrictions on the country of publication and the performance or accuracy of the ViT used in the studies. Finally, for practical reasons, we included studies published only in English.

### Study Selection

Two reviewers, HA and RQ, independently screened the titles and abstracts of the studies retrieved in the search process. In abstract screening, the reviewers excluded the studies that did not fulfill the inclusion criteria. The studies retained after the title and abstract were included for full-text reading. At this stage, disagreements between the 2 reviewers (HA and RQ) were analyzed and resolved through mutual discussion. Finally, the study selection was verified by a third reviewer.

### Data Extraction

We designed a custom-built data extraction sheet. Multimedia Appendix 3 presents the different fields of information in the data extraction sheet. Initially, we pilot-tested the fields in the extraction sheet by extracting data from 7 relevant studies. Two reviewers (HA and RQ) extracted the data from the included studies. The critical information extracted was the application of ViT, the architectures of ViT, the complexity of the architectures used, the pipeline for combining ViT and CNNs, the data sets and their relevant features, and the open research questions identified in the studies. The 2 reviewers resolved disagreements through mutual discussions and revisiting the full text of the relevant study where needed.

### Data Synthesis

We followed a narrative approach to synthesize the data after data extraction. We categorized the included studies based on applications, such as tumor segmentation, grading, or prognosis. We also organized the studies based on data type, such as public versus private data and 2D versus 3D data. We also identified the modality of the data used in the included studies, such as MRI or pathology images. Next, we identified the most frequently used architectures of ViT and the key pipelines for incorporating ViT in cascade or parallel connections with CNN models. We also classified the included studies based on the metrics used to evaluate the performances. Finally, if available, we identified the public code repositories for the model implementation as reported in the included studies.

## Results

### Search Results

**Figure 1:**
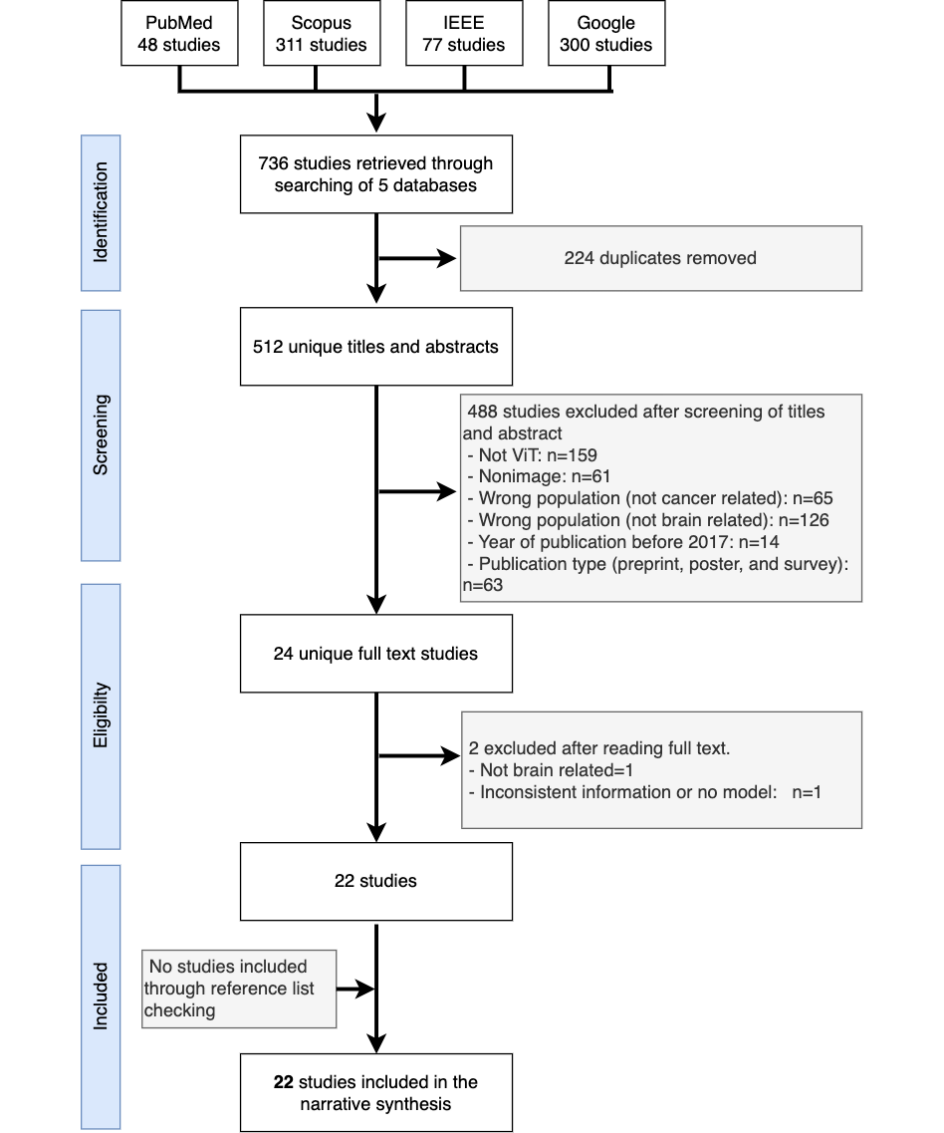
The PRISMA-ScR (Preferred Reporting Items for Systematic Reviews and Meta-Analyses extension for Scoping Reviews) flowchart for the selection of the included studies. ViT: vision transformer.

A total of 736 studies were retrieved. Of these, we removed 224 duplicates. After the title, abstract, and metadata screening, we removed 488 studies that did not fulfill the inclusion criteria and retained 24 studies. In the full-text screening, we removed 2 studies. Overall, 22 studies were included in this review. We did not find any additional studies by forward and backward reference checking. Figure 1 shows the flowchart for the study selection process. Multimedia Appendix 4 shows a list of all the included studies.

### Demographics of the Included Studies

**Figure 2:**
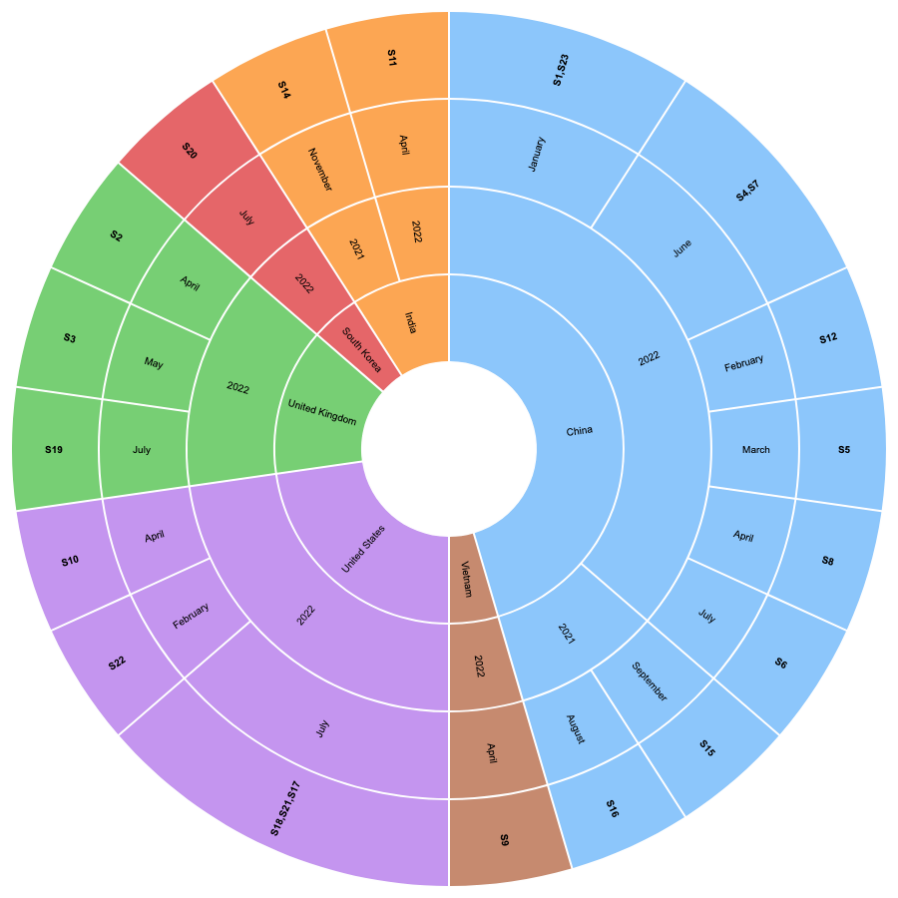
Mapping of the included studies with year, month, and country. S1 through S22 are the included studies.

Among the 22 included studies, 9 (41%) were published in peer-reviewed journals, whereas 13 (59%) were published as conference or workshop proceedings. Of the 22 studies, 19 (86%) were published in 2022, whereas only 3 (14%) were published in 2021. No studies published before 2021 were found. Among the studies published in 2022, one-third (6/22, 27%) were published in July. The included studies were published by authors from 6 different countries (based on first-author affiliation). Among the 22 studies, almost half (n=10, 45%) were published by authors from China and 5 (23%) were published by authors from the United States. Authors from the United Kingdom and India published 3 and 2 studies, respectively, whereas both South Korea and Vietnam published 1 study each. Multimedia Appendix 5 shows a summary of the year-wise and month-wise studies. Multimedia Appendix 6 shows a summary of the country-wise demographics of the included studies. Table 2 summarizes the demographics of the included studies. Figure 2 shows a visualization for the mapping of the included studies with year, month, and country of publication.

**Table 2:** Demographics of the included studies (N=22).

				Studies, n (%)
**Year and month**				
	**2022**			
		January	2 (9)	
		February	2 (9)	
		March	1 (4.5)	
		April	5 (23)	
		May	1 (4.5)	
		June	2 (9)	
		July	6 (27)	
	**2021**			
		August	1 (4.5)	
		September	1 (4.5)	
		November	1 (4.5)	
**Countries**				
	China		10 (45)	
	United States		5 (23)	
	United Kingdom		3 (14)	
	India		2 (9)	
	South Korea		1 (4.5)	
	Vietnam		1 (4.5)	
**Type of publication**				
	Conference		13 (59)	
	Journal		9 (41)	

### Main Tasks Addressed in the Studies

Among the included studies, 19 (86%) of the 22 studies addressed the task of segmentation [27-45], and 1 study [46] reported survival prediction. One study [47] reported the detection of lesions. One study [48] performed grading of the tumor. In addition, 1 study [43] performed the diagnosis of multiple sclerosis, and 1 study [45] performed reconstruction of fast MRI. One study [44] also performed isocitrate dehydrogenase (IDH) genotyping in addition to segmentation. Table 3 shows a summary of the key characteristics and tasks addressed in the included studies.

**Table 3:** Summary of key characteristics of the included studies.

Reference	Year	3D model	2D model	Image modality	Purpose	Transformer name	Data source
[27]	2022	Yes	Yes	MRI^a^	Segmentation	SWIN^b^ transformer	Public
[28]	2022	Yes	No	MRI	Segmentation	SWIN transformer	Public
[29]	2022	Yes	No	MRI	Segmentation	SWIN transformer	Public
[30]	2022	Yes	No	MRI	Segmentation	Not available	Public
[31]	2022	Yes	No	MRI	Segmentation	Segtransvae	Public
[32]	2021	Yes	No	MRI	Segmentation	TransBTS	Public
[33]	2021	Yes	Yes	MRI	Segmentation	SegTran	Public
[34]	2022	Yes	No	MRI	Segmentation	SWIN transformer	Public
[35]	2022	Yes	No	MRI	Segmentation	TransUNet	Public
[36]	2022	Yes	No	MRI	Segmentation	Not available	Public
[37]	2022	Yes	No	MRI	Segmentation	TransBTS	Public
[38]	2022	Yes	No	MRI	Segmentation	UNETR^c^	Public
[39]	2022	Yes	No	MRI	Segmentation	SWIN transformer	Public
[40]	2021	Yes	No	MRI	Segmentation	Not available	Public
[41]	2022	No	Yes	MRI	Segmentation	Not available	Public
[42]	2022	No	Yes	MRI	Segmentation	Not available	Public+private
[43]	2022	Yes	Yes	MRI	Segmentation and diagnosis	Autoregressive transformer	Public
[44]	2022	Yes	No	MRI	Segmentation and grading	Not available	Public
[45]	2022	No	Yes	MRI	Segmentation and reconstruction	SWIN transformer	Public
[46]	2022	No	Yes	MRI	SP^d^	Not available	Public
[47]	2022	No	Yes	MRI	Detection	Not available	Private
[48]	2022	No	Yes	Pathology	Grading	Not available	Private

^a^MRI: magnetic resonance imaging.

^b^SWIN: Shifted Window.

^c^UNETR: UNet Transformer.

^d^SP: survival prediction.

### Key Architectures of the ViT for Brain Tumor Segmentation

In the included studies, ViTs were combined with different variants of a CNN to improve the overall performance of brain tumor segmentation. Shifted Window (SWIN) transformer [49] has recently become a popular choice for image-based classification tasks. Therefore, the most recent studies [27-29,34,39,45] reported using SWIN transformers in their models. Some of the studies [28,29,36,38,40,41] incorporated the transformers module within the encoder or decoder or both modules of the UNet-like architectures. Some studies [30-33,35,37,44] used the transformer module as a bottleneck between the encoder and decoder modules of UNet-like architectures. One study [41] explored both cascade and parallel combinations of the transformer module with CNNs. One study [48] used the transformer module in parallel combination with a residual network (a CNN). One study [42] implemented the training of transformers using federated learning over distributed data for 22 institutions.

### Complexity of the Models Used in the Studies

The included studies presented transformer-based models with different computational complexity. Of these, Fidon et al [35] used the TransUNet model, which has 116.7 million parameters, whereas the UNETR model proposed by Hatamizadeh et al [38] has 92.58 million parameters. The SegTran model proposed by Li et al [33] has 93.1 million parameters. Compared with the UNETR [38], the recent variant, that is, SWIN UNETR [34], has 61.98 million parameters. The Segtransvae [31] has 44.7 million parameters. The BTSWIN-UNet model [28] has 35.6 million parameters that are higher than other SWIN transformer–based models but much smaller than the UNETR. For example, the SWIN transformer–based models Trans-BTS and SWIN-UNet have 30.6 million and 27.1 million parameters, respectively, on the same data, but UNETR has 102.8 million parameters on the same data. The TransConver proposed by Liang et al [27] has 9 million parameters. The SWINMR [45] has 11.40 million parameters for reconstruction. Other studies [28,30,32,36,37,39-44,46-48] did not provide details regarding the computational complexity of the models. Some studies have reported a different number of parameters for other models used on their data. We believe that these minor differences occur because of the resolution of the input images, which may not be the same in different studies.

### Hardware Use

Wang et al [32] used 8 NVIDIA Titan RTX GPUs for training their model. Similarly, Hatamizadeh et al [34] and Hatamizadeh et al [38] trained their models on a DGX-1 cluster with 8 NVIDIA V100 GPUs. Jia and Shu [37] used 4 NVIDIA RTX 8000 GPUs for training the model, whereas Zhou et al [48] used 4 GeForce RTX 2080 Ti GPUs. Liang et al [27] and Liang et al [29] trained their models on 2 parallel NVIDIA GeForce 2080Ti GPUs. Similarly, Huang et al [45] trained the model on 2 NVIDIA RTX 3090 GPUs with 24 GB GPU memory, and Cheng et al [44] used 2 NVIDIA V100 GPUs. Zhang et al [30] and Li et al [47] trained their models on a single NVIDIA Tesla V100 GPU, Li et al [33] trained the model on a single 24 GB Titan RTX GPU, Luu and Park [36] used a single NVIDIA RTX 3090 GPU for training the model, Liu et al [39] trained the model using NVIDIA GTX 3080, and Dhamija et al [41] used Tesla P-100 GPU.

### Types of Data Used in the Studies

**Figure 3:**
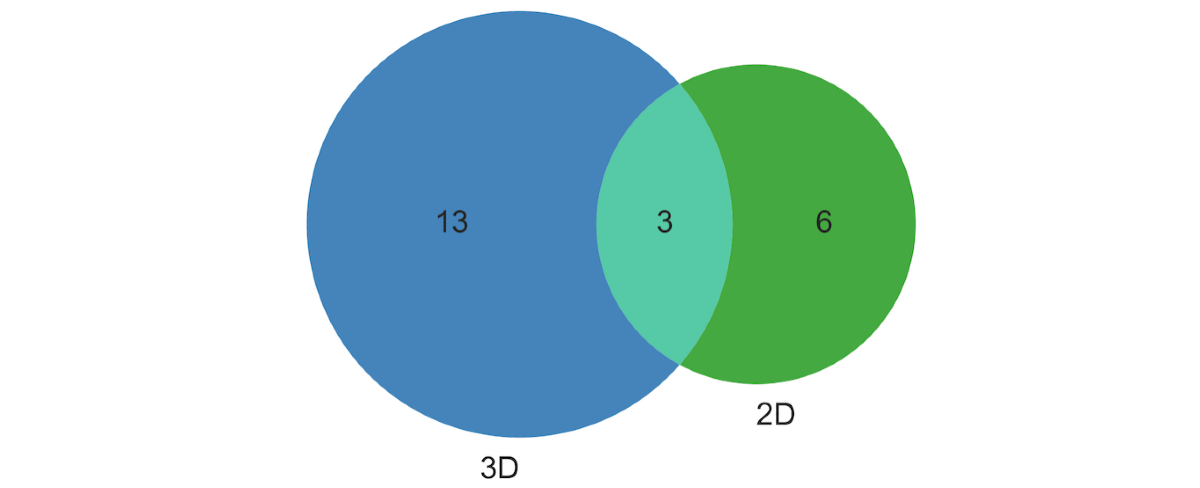
Venn diagrams showing the number of studies that used 3D versus 2D data.

All the included studies (except 1 [48]) used MRI data for brain tumor segmentation. Zhou et al [48] used histopathology images. In 16 studies, volumetric MRI data were used, whereas in 9 studies, the models were developed for 2D image data. Three studies [27,33,43] reported experiments on both volumetric data and image data. Figure 3 shows the Venn diagram for the number of studies using 3D versus 2D data.

### Data Sets Used in the Studies

**Figure 4:**
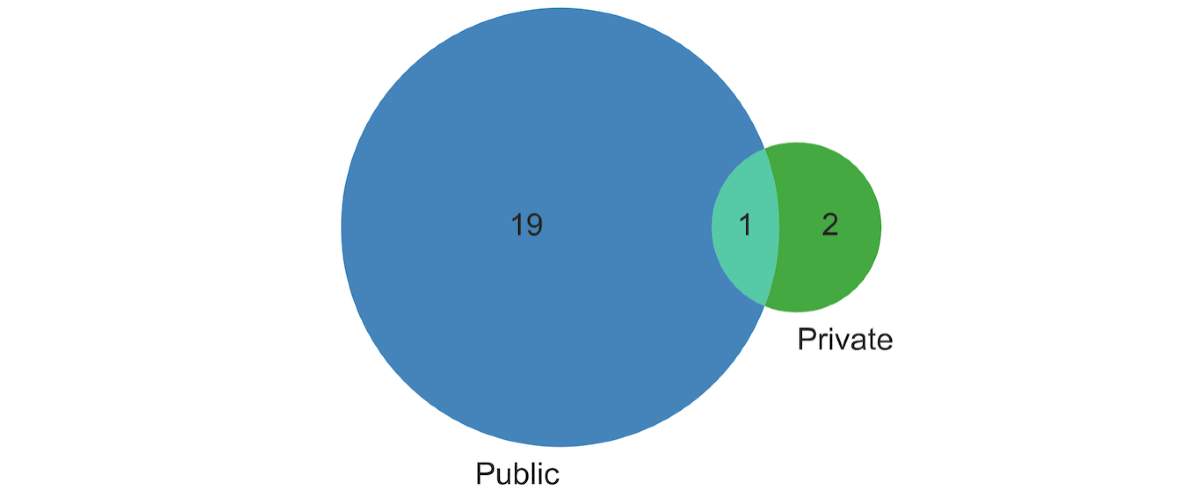
Venn diagrams showing the number of studies that used public versus private data sets.

Three studies [42,47,48] reported using privately developed data sets or did not provide public access to the data. One study [42] used both publicly available and privately developed data. The Brain Tumor Segmentation (BraTS) challenge data set of brain MRI has been the most popular data used in 17 (77%) of the 22 studies. More specifically, 6 studies used BraTS 2021 data [28,31,34-37], 5 used BraTS 2020 data [28,32,42,44,46], 7 used BraTS 2019 data [27-29,32,33,39,40], 3 used BraTS 2018 data [27,29,43], and 1 used BraTS 2017 data [45]. Some of these studies also used >1 data set, either independently or by combining them. Other data used in the included studies were MRI data from the Medical Decathlon used by Hatamizadeh et al [38], the Cancer Imaging Archive data used by Dhamija at [41], the UK Biobank data used by Pinaya et al [43], data from the University Hospital of Ljubljana used by Pinaya et al [43], the Calgary-Campinas Magnetic Resonance reconstruction data used by Huang et al [45], data from the University Hospital of Patras Greece used by Zhou et al [48], and data from the Cancer Hospital and Shenzhen Hospital used by Li et al [47]. One study [30] did not specify the data. Table 4 summarizes the data sets used in the included studies and provides the public access links for each data set. Figure 4 shows the Venn diagram for the number of studies using public versus private data.

**Table 4:** Data sets used in the included studies.

Data set name	Modality	Available	URL	Used by the following studies
BraTS^a^ 2021	MRI^b^	Public	[50]	[28,31,34-37]
BraTS 2020	MRI	Public	[51]	[28,32,42,44,46]
BraTS 2019	MRI	Public	[52]	[27-29,32,33,39,40]
BraTS 2018	MRI	Public	[53]	[27,29,43]
BraTS 2017	MRI	Public	[50]	[45]
Decathlon	MRI	Public	[54]	[38]
TCIA^c^	MRI	Public	[55]	[41]
UK Biobank	MRI	Public	[56]	[43]
University Hospital of Ljubljana	MRI	Public	[57]	[43]
Calgary-Campinas MR^d^ reconstruction data set	MRI	Public	[58]	[45]
University Hospital of Patras Greece	Pathology images	Private	—^e^	[48]
Cancer Hospital and Shenzhen Hospital data	—	Private	—	[47]
Not specified	N/A^f^	N/A	N/A	[30,47]

^a^BraTS: brain tumor segmentation.

^b^MRI: magnetic resonance imaging.

^c^TCIA: The Cancer Imaging Archive.

^d^MR: magnetic resonance.

^e^Not available.

^f^N/A: not applicable.

### Evaluation Metrics

The Dice score and the Hausdorff distance measurements are popular metrics commonly used to evaluate segmentation performance on the BraTS MRI data sets. Hence, in the included studies, the Dice score and Hausdorff distance were the most common metrics used to assess the results of brain tumor segmentation. In summary, 19 studies [27-45] reported the use of the Dice score, whereas 15 studies [27-32,34-40,42,44] used both the Dice score and Hausdorff distance. Two studies [41,45] reported intersection-over-union. One study [42] reported the focal score and Tversky score for the federated learning framework evaluation in addition to the Dice score and Hausdorff distance for the segmentation evaluation. One study [45] reported peak signal:noise ratio, structural similarity index, and Fréchet Inception Distance in the assessment of the reconstructed MRI in addition to Intersection over Union and Dice scores for segmentation evaluation. One study [46] used the concordance index and hazard ratio to evaluate the performance of survival analysis. One study [47] reported sensitivity and precision, and 1 study [48] reported precision and recall.

## Discussion

### Principal Findings

In this study, we reviewed the studies that used ViT to aid in brain cancer imaging applications. We found that most studies (19/22, 86%) were published in 2022, and almost one-third of these studies (6/19, 32%) were published in the second quarter of 2022. As ViT was first proposed in 2020 for natural images, it has only recently been explored in brain MRI and cancer imaging. Almost half of the studies (10/22, 45%) were published by authors from China. Furthermore, the authors from China published twice the number of studies published by authors from the United States. Other countries published approximately one-third of the studies (7/22, 32%).

### Motivation of Using Transformers for Segmentation

The transformer module works on the self-attention concept, that is, calculating pairwise interactions between all input units. Thus, transformers are good at learning contextualized features. Although this learning of the contextualization by a transformer can be related to the upsampling path in a UNet encoder-decoder architecture, the transformer overcomes the limitation of the receptive field, and hence, it works better to capture long-range correlations [34]. In a UNet architecture, one may enlarge the receptive fields by adding more downsampling layers or by introducing larger stride sizes in the convolution operations of the downsampling path. However, the former increases the number of parameters and may lead to overfitting, whereas the latter sacrifices the spatial precision of the feature maps [34]. Nevertheless, the initial attempts to introduce transformers for brain tumor segmentation used the transformer block in the encoder or decoder or the bottleneck stage of the UNet-like architectures. These approaches were mainly driven by the success of UNet-based architectures for segmentation, such as nnUNet’s success on the BraTS2020 challenge [59]. In addition, until 2020, CNN-based models were the best performers for brain tumor segmentation. Therefore, nnUNet [59] was the winning entry for the BraTS2020 challenge. With improved strategies and architectures, attention-based models performed competitively in recent years. Wang et al [32] presented the TransBTS model, which was the first attempt to incorporate transformers into a 3D CNN for brain tumor segmentation. Although Hatamizadeh et al [34] reported SWIN UNETR for brain tumor segmentation, and it was the first transformer-based model that performed competitively for the BraTS 2021 segmentation task. The TransBTS model was trained and tested on the BraTS2018 and BraTS2019 data sets, whereas the SWIN UNETR has been evaluated on the BraTS 2021 data set. However, for the BraTS 2021 data set, the winning entry was an extension of the nnUNet model [59] presented by Luu and Park [36] who proposed introducing attention in the decoder of the nnUNet to perform the tumor segmentation. As identified by Jia and Shu [37], the UNETR removed convolutional blocks in the encoder, which may result in insufficient extraction of local context information when applied to volumetric MRI data. Overall, these approaches of combining transformers and CNNs are driven by the motivation to use the best of both worlds. These studies suggested that the best-of-both-worlds approach can be effective in improving brain tumor segmentation by combining CNNs with transformers. In theory, there are many possibilities for how we approach combining the advantages offered by the 2 different architectures.

### Applications Covered in the Studies

Most of the studies included are those that either designed an attention-based architecture or used existing ViT architectures to achieve the task of tumor segmentation. In the brain segmentation tasks, the key focus is the segmentation of gliomas, which is the most common brain tumor. As most of these studies used 1 of the variants of the BraTS data set where the MRI data are annotated for 4 regions, these studies reported segmentation of the whole tumor, tumor core, enhancing tumor, and background. Some studies also reported using attention-based models for other applications related to brain cancer, such as survival prediction, MRI reconstruction, grading of brain cancer, and IDH genotyping.

### Discussion Related to the Architectures

Among the studies that used the ViT module after a 3D CNN features extraction, the TransBTS [32] was the first architecture (released in September 2021) and served as inspiration for many other architectures. The TransBTS architecture was motivated by the idea of incorporating global context into the volumetric spatial features of brain tumors. Furthermore, the work highlighted the need to use an attention module on image patches instead of flattened images, unlike previous efforts. Essentially, the flattening of high-resolution images makes the implementation impractical, as transformers have a quadratic computational complexity with respect to the number of tokens (ie, the dimension of the flattened image). The TranBTS architecture has downsampling and upsampling layers linked through skip connections; however, in the bottom part of the architecture, there are transformer layers that help with the global context capturing. These transformer layers are in addition to a linear projection layer and a patch embedding layer to transfer the image to sequence representation. So, in a way, the ViT serves as the bottleneck layer to capture long-range dependencies. Later, Jia and Shu [37] presented a modification in the TransBTS architecture [32] using 2 ViT blocks after the encoder part instead of 1 transformer block in the TransBTS. Specifically, the outputs of the fourth and fifth downsampling layers pass through a feature embedding of a feature representation layer, transformer layers, and a feature mapping layer and then pass through the corresponding upsampling 3D CNN layers. Compared with the TransBTS architecture, where the transformer was used at the end of the encoder and features representation was obtained after the fourth layer, Jia and Shu [37], increased the depth to 5 layers and used the transformer in both the fourth and fifth layers. Therefore, after the fourth layer, the transformer effectively builds a skip connection with the corresponding layer of the decoder block.

Similarly, Zhang et al [30] used a multihead self-attention–based transcoder module embedded after the encoder of a 3D UNet. However, they replaced the residual blocks of the 3D UNet with a self-attention layer that operated on a 3D feature map, followed by progressive upsampling via a 3D CNN decoder module. Pham et al [31] also used transformer layers after a 3D CNN module and used a variational encoder to reconstruct the volumetric images. Li et al [33] presented the SegTran architecture, which is again based on using the transformer modules after the features extraction with CNN, thus capturing the global context. Here, the authors suggested combining the CNN features with positional encodings of the pixel coordinates and flattening them into a sequence of local feature vectors.

Fidon et al [35] used the TransUNet architecture [60] as the backbone of their model and used the test time augmentation strategy to improve inference. Finally, Cheng et al [44] presented the MTTUNet architecture, which is a UNet-like encoder-decoder architecture for multitasking. They used the CNN layers to extract spatial features, which were then processed by the bottleneck transformer block. Subsequently, the decoder network performed the segmentation task. In addition, the authors also used the transformer output to perform IDH genotyping, thus making it a multitask architecture.

Hatamizadeh et al [38] presented the UNETR architecture that redefined the task of 3D segmentation as a 1D sequence-to-sequence classification that can be used with a transformer to learn contextual information. Therefore, the transformer block in the UNETR operates on the embedded representation of the 3D MRI input data. In effect, the transformer is incorporated within the encoder part of a UNet architecture. Compared with other architectures such as BTSWIN-UNet [30], TransBTS [32], SegTran [33,35], and BiTr-UNet [37], which use the transformer as a bottleneck layer of the encoder-decoder architectures, the UNETR directly connects the encoded representation from the encoder with the decoder part. Compared with other methods where the encoder part uses 3D CNN blocks, such as TransBTS [32] and BiTr-UNet [37], the UNETR does not use a convolutional block in the encoder. Instead, the UNETR obtains a 2D representation for the 3D volumes and then uses the 2D ViT architecture that works on the 2D patches of the images. Each patch is treated as 1 token for the attention operation. UNETR does not rely on a backbone CNN for generating the input sequences and directly uses the tokenized patches.

Luu and Park [36] introduced an attention mechanism in the decoder of the nnUNet [59] to perform the tumor segmentation. They extended the nnUNet and modified it by using axial attention in the decoder of the 3D UNet. Furthermore, they doubled the number of filters in the encoder while retaining the same number in the decoder. Sagar [40] presented the Vision Transformer for Biomedical Image Segmentation architecture, which used transformer blocks in the encoder and decoder of a UNet architecture. The architecture introduced multiscale convolutions for feature extraction that were used as input to the transformer block.

Dhamija et al [41] explored the sequential and parallel stacks of transformer-based blocks using a UNet block. In principle, they used a transformer-based encoder and a CNN-based decoder connected in parallel with a UNet-based encoder and then in cascade with a UNet-based encoder. Apparently, the parallel combination (USegTransformer-P) outperformed the cascade combination by some margin. Zhou et al [48] designed a parallel dual-branch network of a CNN (the ResNet architecture) and ViT and used it to grade brain cancer from pathology images. The dual-branch network established a duplex communication between the ResNet and ViT blocks that sends global information from the ViT to ResNet and local information from ResNet to the ViT.

Many similar architectures were probably released concurrently by different research groups or released very close in time to each other. For example, Li et al [33] found that segmentation transformer [61] and TransUNet [60] were released concurrently with their own model. Therefore, it is not surprising that there are a few similarities between the approaches adopted by these studies.

### Discussion Related to SWIN Transformers

In general, transformers are notoriously popular for the computational complexity of the order O (n^2^). For example, as identified by Jia and Shu [37], UNETR stacks transformer layers and keeps the sequence data dimension unchanged during the entire process, which results in expensive computation for high-resolution 3D images. SWIN transformers helped overcome the computational complexity. Hence, it became a popular backbone architecture for many recent studies [27-29,32,39,45] to overcome the computational complexity of transformer-based models. For example, Liang et al [27] reported the use of a 2D SWIN transformer [49] and a 3D SWIN transformer [62] to replace the traditional architecture of ViT to overcome the computational complexity. Jiang et al [28] used a SWIN transformer as the encoder and decoder rather than as the attention layer. Furthermore, they extended the 2D SWIN transformer to a 3D variant that provided a base module. Similarly, Liang et al [29] used a 3D SWIN transformer block in the encoder and decoder of a 3D UNet-like architecture. The architecture was inspired by the SWIN transformer and the SWIN-UNet model; however, they replaced the patchify stem with a convolutional stem to stabilize the model training. Furthermore, they used overlapping patch embedding and downsampling, which helped to enhance the locality of the segmentation network.

Hatamizadeh et al [34] extended the UNETR architecture to the SWIN-UNet transformer (SWIN UNETR), which incorporated a SWIN transformer in the encoder part of the 3D UNet. The decoder part still used a CNN architecture to upsample the features to the segmentation masks. As reported previously, the SWIN UNETR was the first transformer architecture that performed competitively on the BraTS 2021 segmentation challenge. Liu et al [39] presented a transition net architecture that combined a 2D SWIN transformer with a 3D transition decoder. The transition block transforms the 3D volumetric data into a 2D representation, which is then provided as an input to the SWIN transformer. Subsequently, in the decoder part, the transition block transforms the multiscale feature maps into a 3D representation to obtain the segmentation results. Huang et al [45] used a cascade of residual SWIN transformers to build a feature extraction module, followed by a 2D CNN network. This architecture was designed for MRI reconstruction.

### Discussion Related to Model Complexity

In general, transformer architectures have a high computational complexity. The number of parameters for the architectures for the models, such as UNETR and TransUNet, are as large as 92 million and 116 million, respectively. The SWIN transformer-based architecture has a relatively smaller number of parameters (of the order of 30-45 million). For models with a higher number of parameters, the researchers had to rely on high-end GPU resources. Therefore, the computational setup reported in some of the included studies was built with as many as 8 GPUs. However, few studies also reported training the models on a single GPU with memory sizes ranging from 12 GB to 24 GB.

### Discussion Related to 3D Data

Our categorization of a model designed for 3D or 2D data was either based on direct extraction of the information from the studies or the description of the model architecture in the included studies. Therefore, if a study did not specify whether it used the volumetric data directly or transformed the data into 2D images but provided a 2D model architecture, we placed the study in the 2D data category. Many modern deep learning methods for medical imaging, including transformers, rely on pretrained models as their backbones. These backbones can generalize well, making them good candidates for use in other related tasks, as they provide generalization, better convergence, and improved segmentation performance [39]. However, Liu et al [39] argued that such backbone architectures are, in general, difficult to be migrated to 3D brain tumor segmentation. First, there is a general lack of 3D data, and most publicly available data sets provide 2D data. Second, medical images such as MRI vary in their distribution and style compared with natural images. These variations hinder the direct transformation of the 2D pretrained models for 3D volumetric data. Hence, they recommended transforming the 3D data into a 2D representation to enable its use with 2D transformers. However, numerous other studies have developed and used 3D models directly on volumetric data.

The most commonly used data in the included studies were the brain MRI of the BraTS data set. The BraTS data set has been phenomenal in facilitating the research on brain glioma segmentation. The BraTS challenge has served as a dedicated venue for the last 11 years and has established itself as a foundation data set in helping the community push the state-of-the-art in brain tumor segmentation. The BraTS data set has 4 MRI modalities, namely, T1-weighted, postcontrast T1-weighted, T2-weighted, and T2 fluid-attenuated inversion recovery. Furthermore, the data set provides baseline segmentation annotation from physicians.

### Discussion Related to Evaluation Metrics

The Dice score and Hausdorff distance measurements have been more commonly reported, as these metrics are widely used to evaluate segmentation performance on the BraTS MRI data sets. In the included studies, the Dice score and Hausdorff distance were the most common metrics used to assess the results of brain tumor segmentation.

### Strengths and Limitations

#### Strengths

Although there has been a surge in studies on the use of ViTs in medical imaging, only a few reviews have been reported on ViTs in medical imaging [20,23,25]; however, their scopes are too broad. In comparison, to the best of our knowledge, this is the first review of the applications and potential of ViTs to enhance the performance of brain tumor segmentation. This review covers all the studies that used ViTs for brain cancer imaging; thus, this is the most comprehensive review. This review is helpful for the community interested in knowing the different architectures of ViTs that can help in brain tumor segmentation. Unlike other reviews [20,23,25] that cover many different medical imaging applications, this review focuses on studies that have only developed ViTs for brain tumor segmentation. In this review, we followed the PRISMA-ScR guidelines [26]. We retrieved articles from the popular web-based libraries of medical science and computing to include as many relevant studies as possible. We avoided bias in study selection through an independent selection of studies by 2 reviewers and through validation of the selected studies and data extraction by the third reviewer. This review provides a comprehensive discussion on the different pipelines to combine ViTs with CNNs. Hence, this review will be very useful for the community to learn about the different pipelines and their working for brain tumor segmentation. In addition, we identify the computational complexity of the various pipelines to help the readers understand the associated computational cost of ViTs for brain tumor segmentation. We provide a comprehensive list of available data sets for brain MRI and hope that it will provide a good reference point for researchers to identify suitable data sets for developing models for BraTS. We maintain an active web-based repository that will be populated with relevant studies in the future.

#### Limitations

In this review, we included studies from 4 major databases. Despite our best efforts to retrieve as many studies as possible, the possibility that some relevant studies may be missed cannot be ruled out. Moreover, the number of publications on the applications of ViTs in medical imaging is increasing at an unprecedented rate; hence, recent studies may be published while we draft this work. For practical reasons, we only included studies in English. Therefore, non-English text might be excluded even if it were relevant. Not all studies reported on the computational complexity and the required training time. Hence, we provide the computational complexity only for the studies in which this information was available; thus, the comparison might not be exhaustive. This review did not analyze the claims on the performance of the different architectures, as such an assessment is beyond the scope of this work. We did not attempt to reproduce the results reported in the studies, as such an execution of the computer code is beyond the scope of the review. We included studies that reported working with any imaging modality for brain cancer and did not evaluate the use of physiological signals, although understanding physiological signals can also play a significant role in brain cancer studies. We did not evaluate the bias in the training data used in the included studies; therefore, the performance reported for ViTs in brain cancer imaging could be occasionally overestimated.

### Open Questions and Challenges

Research efforts on developing transformer-based methods for brain cancer applications are progressing rapidly. Some of the challenges are highlighted in the following text.

In the included studies, we did not find any study that addresses the challenge of early detection of brain cancer. Similarly, the number of studies related to prognosis and tumor growth in the brain is also minimal. Early detection and prognosis are applications of great interest where the potential of ViTs can be explored. One approach is to combine ViT with the sequential representation of time-based data for tumor growth in the brain.

ViTs lack scale invariance, rotation invariance, and inductive bias capabilities. Consequently, they do not perform well at capturing local information and cannot be trained well with a small amount of data [48]. One way to overcome this limitation is to provide a larger training data set. Therefore, the development of large public data sets is encouraged. Another widely used method in the included studies is combining ViTs with CNNs.

In general, models pretrained on a large-scale data set (ImageNet) are known to perform well on many other data sets. However, using the pretrained transformer-based models and fine-tuning them for brain cancer imaging did not improve the performance, as reported by Hatamizadeh et al [38]. Similarly, Pinaya et al [43] reported that the model trained on 3D data from the UK Biobank could perform well on the test set. However, the performance degraded when the model was evaluated on subsets of other data sets. Therefore, the generalization of the models is still a challenge.

Combining CNN with ViTs can be achieved through serial (cascade), parallel connections, or a combination of both. In serial combination of CNNs and ViTs, the arrangement may cause training ambiguities in terms of fusing local and global features. If the learning eventually loses local and global dependencies in the image data [48,63,64], optimal performance may not be achieved. In contrast, for parallel combinations, there will be undesired redundant information captured by the 2 models [33].

The BraTS challenge completed its 10 years in 2021 and has been a dedicated venue for facilitating the state-of-the-art developments of methods for glioma segmentation [37]. As the data set is publicly available, almost all the included studies have used it. However, there seems to be a very limited effort in developing other data sets that are publicly available. It would be interesting to have additional data sets for brain cancer imaging that can facilitate advancing the research on AI models for brain cancer diagnosis and prognosis.

The included studies reported advancements in transformer-based architectures for brain cancer imaging. However, these studies commonly lack the explanability and interpretability of the model behavior. Future research should focus on new methods to address this issue.

ViT-based architectures, as of now, may not always be the best for brain tumor segmentation. For example, the TransBTS model (a ViT-based model) had suboptimal performance owing to its inherently inefficient architecture, where the ViT is only used in the bottleneck as a stand-alone attention module and does not have a connection to the decoder at different scales (as identified by Hatamizadeh et al [34]). In contrast, architectures based on UNet (eg, nnUNet and SegResNet) have achieved competitive benchmarks on the BraTS challenge.

As identified by Huang et al [45], one can argue that the heavy computations in transformers are the main bottleneck in development, and the performance improvements of transformers for brain cancer imaging come at the cost of computational complexity. Therefore, lightweight implementations of transformer architectures for brain cancer imaging are a topic of great interest for future research. Furthermore, the transformer architectures that transform image data into sequential representation (such as in UNETR) may not be the best choice. First, the removal of convolutional blocks in the encoder does not guarantee the capture of context information in volumetric MRI data. Second, keeping a fixed sequence during the entire processing of data leads to expensive computation when the input data are a batch of high-resolution 3D images [37]. Models such as UNETR and TransBTS for brain tumor segmentation lack cross-plane contextual information; hence, the 3D spatial context is not fully captured by these models [29].

### Conclusions

In this work, we performed a scoping review of 22 studies that reported ViT-based AI models for brain cancer imaging. We identified the key applications of ViTs in developing AI models for tumor segmentation and grading. ViTs have enabled researchers to push the state-of-the-art in brain tumor segmentation, although such an improvement has resulted in a trade-off between model complexity and performance. We also summarized the different vision architectures and the pipelines with ViTs as the backbone architecture. We also identified the commonly used data sets brain tumor segmentation tasks. Finally, we provided insights into the key challenges in advancing brain cancer diagnosis or prognosis using ViT-based architectures. Although ViT-based architectures have great potential in advancing AI methods for brain cancer, clinical transformations can be challenging, as these models are computationally complex and have limited or no explainability. We believe that the findings of this review will be beneficial to the researchers studying AI and cancer.

## Appendix

Multimedia Appendix 1 PRISMA-ScR (Preferred Reporting Items for Systematic Reviews and Meta-Analyses extension for Scoping Reviews) checklist.

Multimedia Appendix 2 Search terms.

Multimedia Appendix 3 Extraction fields.

Multimedia Appendix 4 Included studies.

Multimedia Appendix 5 Demographics of the included studies showing month-wise publications.

Multimedia Appendix 6 Demographics of the included studies showing country-wise publications.

## Abbreviations

AI: artificial intelligence
BraTS: Brain Tumor Segmentation
CNN: convolutional neural network
GPU: graphics processing unit
IDH: isocitrate dehydrogenase
MRI: magnetic resonance imaging
PRISMA-ScR: Preferred Reporting Items for Systematic Reviews and Meta-Analyses extension for Scoping Reviews
ViT: vision transformer
