# Supplementary material for: Artificial Intelligence–Based Methods for Integrating Local and Global Features for Brain Cancer Imaging: Scoping Review
Source: JMIR Med Inform. 2023 Nov 17;11:e47445. doi: 10.2196/47445 (PMC10692876; doi:10.2196/47445)
Supplement: Multimedia Appendix 2 [file medinform_v11i1e47445_app2.docx]

**Appendix 2: Search Strategy**

Database(s): IEEE Xplore, Pubmed, Scopus and Google Scholar.

Years: 2017-2022

Search dates: 31 July 2022 – 01 August 2022

Initial raw search results: 736

| **Database** | **Search strategy** | **Hits** |
| --- | --- | --- |
| IEEExplore | ((Vision Transformers) OR (Transformers)) AND ((Cancer) OR (cancer imaging) OR (tumor) OR  ( retinoblastoma ) OR (thyroid) OR (leukemia) OR (melanoma) OR (lymphoma) OR (prostate) OR (renal cancer)) | 77 |
| Pubmed | ((Vision Transformers[Title/Abstract]) OR (Transformers[Title/Abstract])) AND ((Cancer[Title/Abstract]) OR (cancer imaging[Title/Abstract]) OR (tumor[Title/Abstract]) OR (Retinoblastoma[Title/Abstract]) OR (thyroid[Title/Abstract]) OR (leukemia[Title/Abstract]) OR (melanoma[Title/Abstract]) OR (lymphoma[Title/Abstract]) OR (prostate[Title/Abstract]) OR (renal cancer[Title/Abstract])) | 48 |
| Scopus | TITLE-ABS-KEY ( ( ( vision AND transformers ) OR ( transformers ) ) AND ( ( cancer ) OR ( cancer AND imaging ) OR ( tumor ) OR ( retinoblastoma ) OR ( thyroid ) OR ( leukemia ) OR ( melanoma ) OR ( lymphoma ) OR ( prostate ) OR ( renal AND cancer ) ) ) AND ( EXCLUDE ( DOCTYPE , "re" ) OR EXCLUDE ( DOCTYPE , "le" ) OR EXCLUDE ( DOCTYPE , "cr" ) ) AND ( LIMIT-TO ( PUBYEAR , 2022 ) OR LIMIT-TO ( PUBYEAR , 2021 ) OR LIMIT-TO ( PUBYEAR , 2020 ) OR LIMIT-TO ( PUBYEAR , 2019 ) OR LIMIT-TO ( PUBYEAR , 2018 ) OR LIMIT-TO ( PUBYEAR , 2017 ) ) AND ( LIMIT-TO ( LANGUAGE , "English" ) ) | 311 |
| Google Scholar | ((Vision Transformers) OR (Transformers)) AND ((Cancer) OR (cancer imaging) OR (tumor) OR ( retinoblastoma ) OR (thyroid) OR (leukemia) OR (melanoma) OR (lymphoma) OR (prostate) OR (renal cancer)) | 300 |
