# Supplementary figures and images for: Artificial Intelligence–Based Methods for Integrating Local and Global Features for Brain Cancer Imaging: Scoping Review
Source: JMIR Med Inform. 2023 Nov 17;11:e47445. doi: 10.2196/47445 (PMC10692876; doi:10.2196/47445)

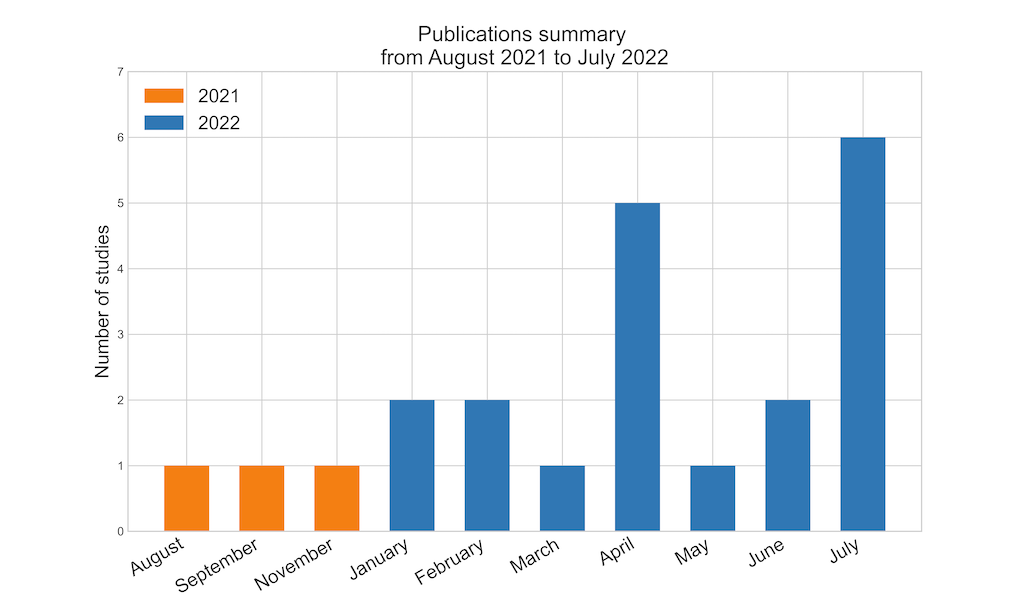

Supplement: Multimedia Appendix 5 [file medinform_v11i1e47445_app5.png]

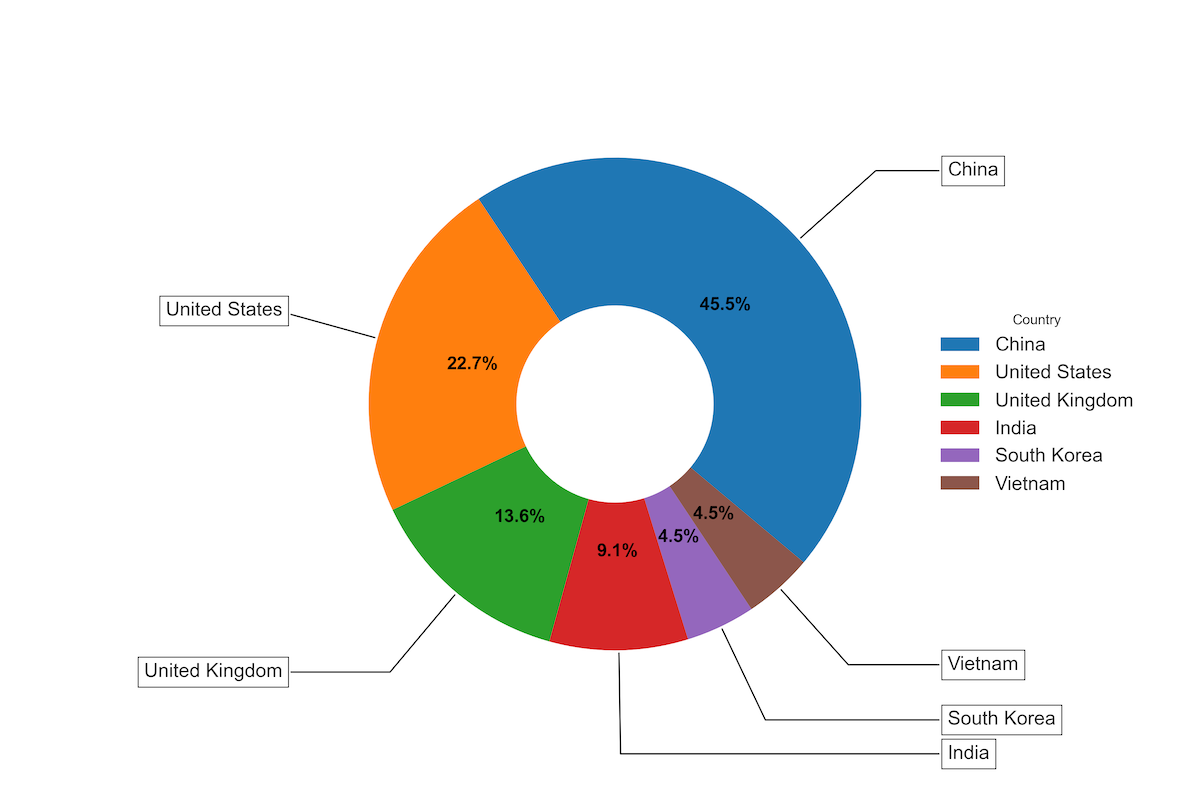

Supplement: Multimedia Appendix 6 [file medinform_v11i1e47445_app6.png]
